# Retraction Note: Screening of feature genes related to immune and inflammatory responses in periodontitis

**DOI:** 10.1186/s12903-024-04378-4

**Published:** 2024-05-29

**Authors:** Azhu Duan, Yeming Zhang, Gongjie Yuan

**Affiliations:** 1 Department of Stomatology, Children’s Hospital of Shanghai, Children’s Hospital Affiliated to Shanghai Jiao Tong University School of Medicine, 1400 Beijing West Road, Jing’an District, Shanghai, 200000 China; 2 Department of Stomatology, Tong Ren Hospital, Shanghai Jiao Tong University School of Medicine, Shanghai, 200000 China

**Retraction Note: BMC Oral Health (2023) 23:234**

10.1186/s12903-023-02925-z

The editor has retracted this article after concerns were raised about the data reported. The two datasets used in this analysis, GSE10334 and GSE16134, were each generated from the same archive of healthy and diseased gingival tissues, and as such there is a significant sampling overlap between the two. By using GSE10334 as a validation dataset for GSE16134, the authors do not appear to have accounted for these limitations. The editor no longer has confidence in the reliability of the article’s results and conclusions.

The authors did not respond to correspondence from the publisher about this retraction.

